# The Utility of Near-Infrared Fluorescence and Indocyanine Green During Robotic Pulmonary Resection

**DOI:** 10.3389/fsurg.2019.00047

**Published:** 2019-08-09

**Authors:** Dana Ferrari-Light, Travis C. Geraci, Prabhu Sasankan, Robert J. Cerfolio

**Affiliations:** ^1^Department of Cardiothoracic Surgery, New York University Langone Health, New York, NY, United States; ^2^School of Medicine, New York University Langone Health, New York, NY, United States

**Keywords:** electromagnetic, fluorescence, localization, navigational bronchoscopy, lung cancer, pulmonary resection, robotic

## Abstract

During minimally invasive pulmonary resection, it is often difficult to localize pulmonary nodules that are small (<2 cm), low-density/subsolid on imaging, or deep to the visceral pleura. The use of near-infrared fluorescence (NIF) imaging for localizing pulmonary nodules using indocyanine green (ICG) contrast is an emerging technology that is increasingly utilized during pulmonary resection. When administered via electromagnetic navigational bronchoscopy (ENB), ICG can accurately localize pulmonary nodules. When injected intravenously (IV), ICG can also help delineate the intersegmental plane. Research is ongoing regarding the utility of ICG for identification of the sentinel lymph node in lung cancer.

## Introduction

The use of electromagnetic navigational bronchoscopy (ENB) using near-infrared fluorescence (NIF) with indocyanine green contrast (ICG) has emerged as an accurate and efficient method for localizing pulmonary nodules. Indocyanine green is a fluorophore contrast that illuminates in the near infrared spectrum, has a high signal-to-noise ratio, is inexpensive, and relatively non-toxic (although the potential for patient allergy remains) (1). Indocyanine green is approved by the U.S. Food and Drug Administration as an intravenously (IV) injected drug for angiography studies and sentinel node assessments in various cancers. It is theorized that ICG is retained in malignant tissue by a non-specific inflammatory permeability and retention effect. Importantly, infiltration with ICG will not distort the cellular integrity of tissues and therefore will not interfere with the histopathologic characterization of the target specimen, including tumor margins.

Indocyanine green has been used successfully in many areas of medicine including ophthalmic angiography, cerebral perfusion assessment, and for the identification of sentinel lymph nodes and evaluation of anastomotic perfusion in various oncologic resections. However, its utility in pulmonary resection has only recently been explored.

In this review we discuss the multimodal use of ICG during pulmonary resection—transbronchial injection for localization of the primary tumor, delineation of the intersegmental plane, and identification of regional lymph nodes.

## Intraoperative Localization of Pulmonary Nodules With Indocyanine Green

The management of pulmonary nodules has evolved in synchrony with technological advancements in chest imaging. The increased precision of modern helical computed tomography (CT) has led to the detection of small pulmonary lesions, ostensibly creating a window of opportunity for curative surgical resection in patients with early stage lung cancer. In 2011, the National Lung Screening Trial (NLST) reported a 20% relative reduction in mortality from lung cancer in high-risk patients screened with low-dose CT (LDCT) rather than chest radiography (2). Now with an exponentially-increasing rate of patients with screening LDCT scans revealing small, ill-defined pulmonary lesions, they increasingly are referred to thoracic surgeons for management. However, the majority of these indeterminate pulmonary nodules do not represent lung cancer. In the NLST, 96.4% of identified lesions were determined to be false-positive results after three screening rounds (2). These patients are continually followed with serial imaging. A minority of patients with more concerning sequential imaging findings require an invasive biopsy or surgical resection for diagnosis and/or definitive treatment.

The current standard of care for patients with small early-stage non-small cell lung cancer (NSCLC) is pulmonary lobectomy (3). This standard, however, is based on data that is over 20 years old, prior to the routine use of preoperative positron emission tomography scans (PET/CT) and the ascendance of minimally-invasive surgical techniques. Sublobar resection such as anatomic segmentectomy is performed more often in patients with small nodules or reduced cardiopulmonary function. Data regarding the efficacy of lobectomy vs. sublobar resection are mixed. Prospective non-randomized data have shown comparable long-term survival in patients with nodules <2 cm without nodal metastasis (4). Two prospective, randomized clinical trials, the Cancer and Leukemia Group B Trial 140503 and the Japan Clinical Oncology Group 0802/WJOG 4607L and JCOG 1211 Trial, are currently being conducted to help address the oncologic superiority or equality of sublobar resection vs. lobectomy, particularly for patients with small, early-stage lung cancer (5).

During sublobar resection, indeterminate lesions are often difficult to localize, particularly when a minimally invasive approach is performed. Nodule characteristics may also make intraoperative localization more difficult, such as when the lesion is smaller than 2 cm, subsolid and/or of low-density on CT scan, or deep to the visceral pleural surface. If performing a segmental resection, the precise location of the lesion may be difficult to determine on CT imaging alone, particularly if it is located between adjacent segments. Additionally, when performing pulmonary resection with robotic assistance, identifying lesions can be difficult due to the system's lack of haptic feedback and reliance on visual cues. These limitations of surgical technique have prompted the development of intraoperative localization techniques to aid in accurate resection. Additional incisions, conversion to thoracotomy for bimanual palpation, or performing a lobectomy to resect an intraoperative non-identifiable lesion are inefficient and morbid strategies. Intraoperative localization avoids these maneuvers and allows for more precise surgery.

Minimally invasive approaches to pulmonary resection are preferred to reduce perioperative morbidity and length of stay, precluding the standard technique of bimanual palpation. A number of preoperative procedures have been developed to aid localization, including CT-guided placement of metallic wires, coils, or markers (6, 7). While these techniques may be accurate, they require coordination with radiology and/or a hybrid operating room, exposes the patient and staff to radiation, requires a significant amount of time to complete, risk migration of the marker, and may be complicated by parenchymal hematoma or pneumothorax.

In 2001, Sakamoto et al. were the first to describe a method for nodule localization using indigo carmine contrast injected via flexible bronchoscopy near the target lesion (8). This study was followed by a series of studies reporting accurate localization of small pulmonary nodules and regional lymph nodes using electromagnetic navigation bronchoscopy (ENB) with transbronchial injection of blue dyes, most commonly, methylene blue or isosulfan blue (9). While the accuracy of these agents range from 79 to 100%, contrast dyes such as indigo carmine and methylene blue are limited by relatively quick diffusion time thereby limiting precision intraoperative visualization. Additionally, unlike color dyes visualized by white light endoscopy, ICG fluorescence is always detectable regardless of color or texture variation in the pulmonary parenchyma such as regions of anthracotic pigmentation, hematoma, or architectural distortion due to underlying pulmonary disease.

In 2015, Anayama et al. confirmed the feasibility of using NIF for lesion localization via ENB-guided ICG injection in a porcine lung model (10). Both *in vitro* and *ex vivo* studies were performed to assess the tissue penetration and spread of ICG in pulmonary parenchyma and to evaluate intraoperative localization. In the same year, Keating et al. published a case report in a human patient of successful intraoperative localization using NIF imaging with ICG during video-assisted thoracoscopic surgery (VATS) (11). In this study, the patient received an intravenous injection of ICG 24 h prior to surgery. Two lesions were successfully visualized and resected, one of which could not be identified with manual palpation through the thoracoscopic port sites.

A number of groups have since reported institutional studies regarding the accuracy of ENB-guided NIF using ICG for intraoperative localization of pulmonary lesions. In 2015 Okusanya et al. reported localization after administering preoperative intravenous ICG (5 mg/kg) followed by open thoracotomy within 24 h (12). NIF detected 16/18 (88%) nodules compared to manual palpation, which achieved a 100% identification rate. Furthermore, ICG identified five additional malignant subcentimeter nodules not readily apparent on CT imaging, three of which were in different lobes than the primary tumor. The authors found that the sensitivity for detecting nodules with NIF was dependent on tissue depth but independent of nodule size, metabolic activity, histology, or vascularity.

In 2017, Abbas et al. reported their institutional experience with ENB, localizing 54 nodules in 51 patients with a success rate of 98.1% and a false negative rate of 1.9% (13). In the first two patients, methylene blue dye with fiducial markers were used however the markers were found to be of marginal utility and the blue dye diffused quickly, prompting the researchers to switch to an combination of iopamidol (for fluoroscopic identification), methylene blue (for visual identification), and ICG (for fluorescence identification). Two patients required conversion to open thoracotomy for localization and resection due to dense pleural adhesions.

In 2018, Anayama et al. reported a series of 37 patients with nodules <2 cm in size who underwent VATS wedge resection using a mixture of ICG and iopamidol for localization by either CT-guided percutaneous injection or bronchoscopic injection (14). In the CT-guided group, 15/15 (100%) nodules were successfully localized, however a small pneumothorax occurred in three patients (20%) and in one patient this precluded additional marking. In the bronchoscopic group, 20/22 (90.9%) nodules were localized with six patients receiving injections at two anatomic locations. In the two patients unable to be localized, finger palpation was successful after extension of the access port site. The authors concluded that CT-guided localization might not be optimal for patients with multiple nodules, as the risk of pneumothorax may prevent marking of multiple lesions. Additionally, the bronchoscopic method may be preferred for patients with nodules that may be difficult to reach percutaneously, such as lesions adjacent the mediastinum, or posteriorly behind the scapula.

In mid-2019, Chao et al. just recently published their experience with a dual-marker localization technique in a hybrid operating room, using NIF marking with ICG in conjunction with microcoil deployment via CT-guided coaxial needle technique. This dual-marker placement was successful in all 11 patients studied, with a median localization time of 19 min and may be helpful to locate difficult pulmonary nodules (15).

We have recently published our own institutional experience with NIF localization using ICG when administered both via ENB and intravenous injection (16). In our series of patients who underwent planned robotic segmentectomy, 93 were selected for ENB localization with ICG due to small nodule size and/or challenging anatomic location (between segments or deep to the visceral pleural surface). Of the 93 patients undergoing ENB, we successfully identified the pulmonary nodule in 80 patients (86%). We were unable to find a statistically significant variable that would predict success or failure of our method. The most common reasons for localization failure were inaccurate ENB or ICG injection and technical malfunctions of the equipment.

## Technical Considerations for ICG Localization

At our institution, the decision to use ENB and ICG fluorescence for pulmonary tumor localization is selective. We chose to localize lesions based on size (favored for small lesions <2 cm), lesion morphology (ground-glass opacification), and lesion anatomy (favored in lesions deep to the pleural surface). There is no distance from the pleura that we consider to be an absolute contraindication to lesion localization.

**Figure 1:**
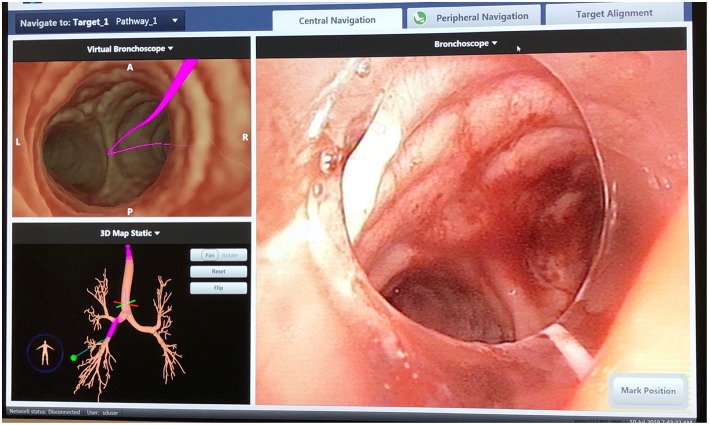
SuperDimension Thoracic Navigation System: electromagnetic bronchoscopic view, showing the pathway to the target nodule in the right lower lobe.

**Figure 2:**
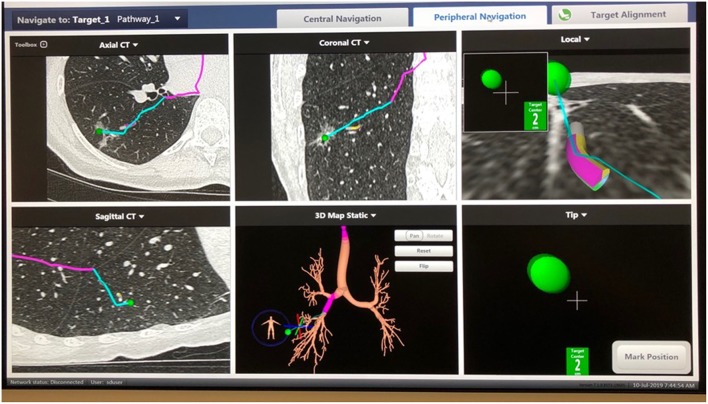
SuperDimension Thoracic Navigation System: image-guided peripheral view, showing electromagnetic probe is 2 cm from the target nodule in the right lower lobe.

Performing localization starts with a preoperative review of collimated CT scan data using superDimension™ software (Covidien, Minneapolis, MN) to create a virtual bronchoscopic pathway to the target lesion (see Figure 1). Identification of the optimal adjacent segmental and/or subsegmental bronchus is important for successful localization. In the operating room, the patient is positioned supine on the operating table and an electromagnetic board is placed beneath. Precordial sensors are attached to the patient which are then registered with the electromagnetic system. The patient is intubated with a single-lumen endotracheal tube. A sensor probe is introduced through the working channel of a flexible bronchoscope and the system is synced with various anatomic coordinates in the proximal and distal airways. Real-time virtual-guided bronchoscopy is then performed to within a goal distance of 1 cm or less to the target lesion (see Figure 2). The flexible probe may be extended beyond the end of the bronchoscope, allowing transparenchymal navigation to peripheral lesions and/or those distant from a subsegmental bronchus. In our experience, upper lobe lesions are the most difficult to localize because of the acute bronchoscopic angles required to reach the apical airways. When the lesion is localized, ICG contrast is injected via a bronchoscopic needle. It is prudent to have an assistant hold the bronchoscopic in position during injection to prevent drifting from the target location.

**Figure 3:**
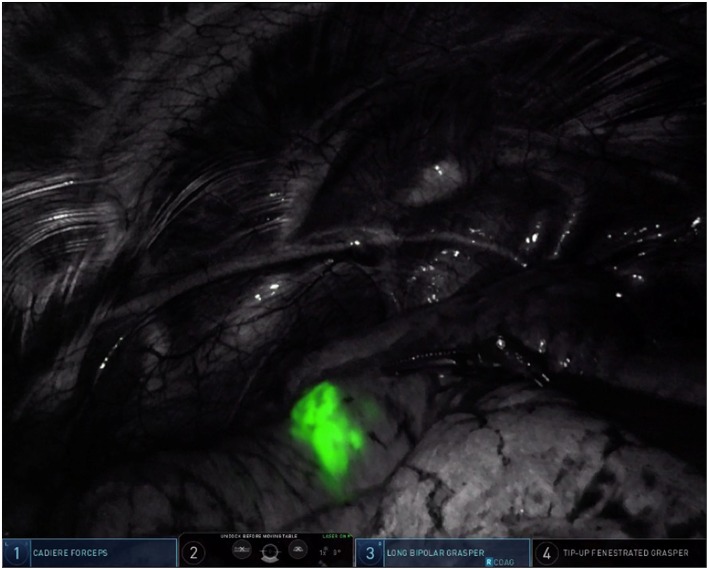
Pulmonary nodule in the left upper lobe, illuminated by ICG fluorescence (firefly mode).

**Figure 4:**
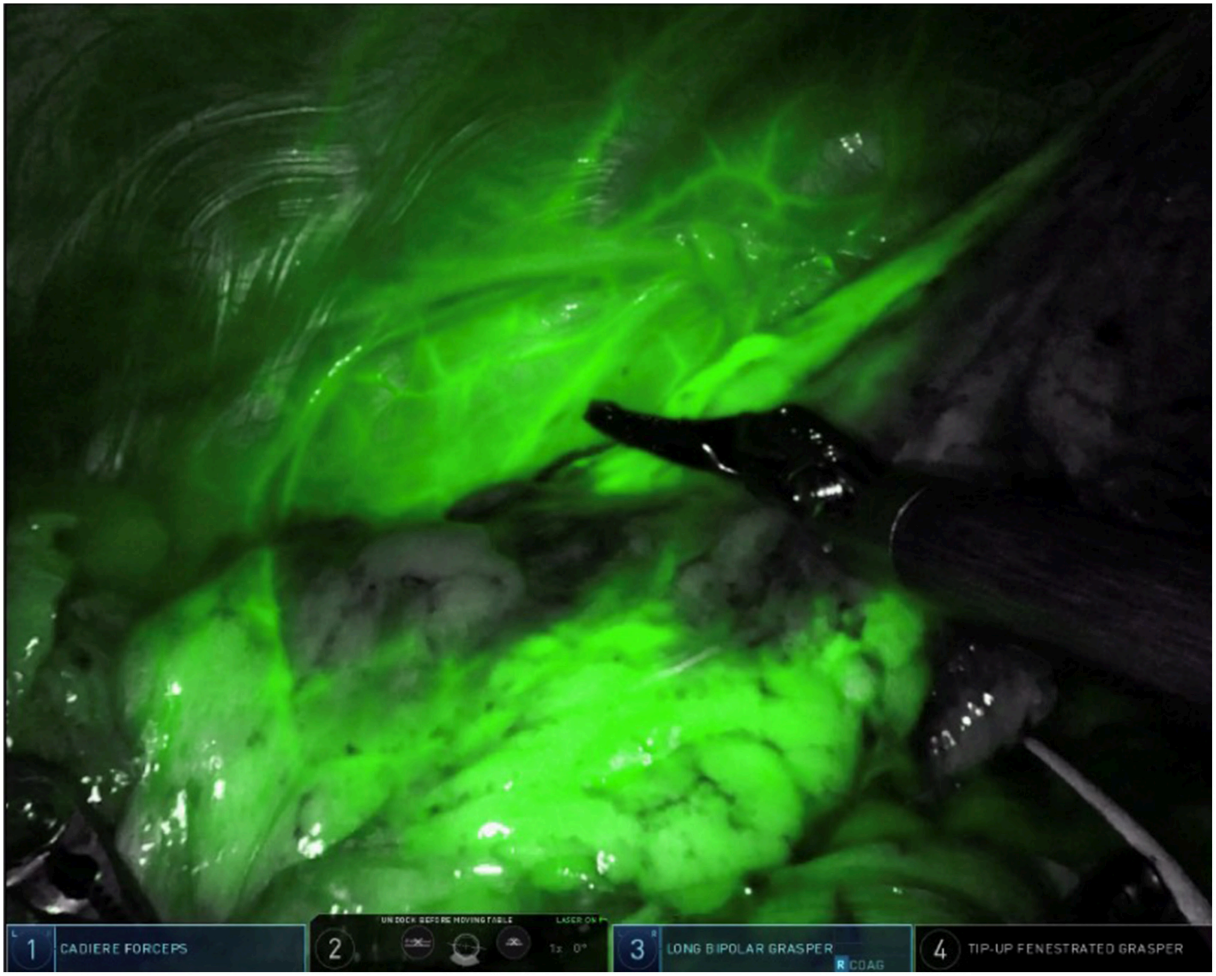
Intravenous ICG delineation of the intersegmental plane between the lingula and the left upper lobe segments during left upper lobe trisegmentectomy. Note the diffusion of ICG contrast around the area of target nodule from Figure 3.

Our method for contrast injection involves an admixture of 10 mL of sterile water in a 25 mg bottled powder of indocyanine green. We inject 0.5 mL of ICG solution followed by a flush of 0.5 mL of sterile water. It is important to use sterile water in the admixture. In our experience, using normal saline will cause the ICG powder to clump and makes it difficult to flush intravenously. The remaining 9.5 mL of ICG solution is given intravenously by the anesthesiologist after control and ligation of the segmental pulmonary artery. During robotic surgery on the Xi version of the da Vinci robotic system, the thoracoscopic camera is equipped with near-infrared technology (Firefly, Intuitive Surgical, Sunnyvale, CA) which illuminates ICG-infiltrated lung parenchyma after peritumoral injection (see Figure 3) and ICG-perfused tissue after intravenous administration (see Figure 4).

## Delineation of the Intersegmental Plane

During anatomic segmental resection, the corresponding vein, artery, and bronchus are transected and the lung parenchyma is divided with a surgical stapler. During parenchymal division, the surgeon must identify the intersegmental plane while achieving an appropriate tumor margin. Standard techniques for identifying the intersegmental plane such as inflation/deflation of the tissue, are practically cumbersome and may be misleading due to air diffusion through the communicating pores of Kohn. Indocyanine green binds to plasma proteins and when injected intravenously is confined to the vascular compartment. Due to this biochemical property, ICG helps delineate the intersegmental plane after ligation of the segmental artery.

Presented in 2017, Mehta et al. conducted a phase II prospective cohort trial evaluating the safety, feasibility, and reproducibility of peripheral venous ICG injection and NIF mapping for delineation of the intersegmental plane during robotic segmental resection (17). Specifically, the researchers assessed whether this technique would increase the oncological margin vs. reliance on visual delineation of the intersegmental plane. In 31 patients who underwent pulmonary resection with ICG injection, 23 (74%) cases resulted in a significant lengthening of the oncologic resection margin. Using a novel 7-point objective rating scale, fluorescent segmental demarcation resulted in an average increase in the oncologic margin of 2.4 cm vs. the predicted plane by a two-surgeon assessment. The long-term significance regarding local control, recurrence, and disease-free survival are unknown, but long-term follow-up is anticipated.

In their 2019 report, Sekine et al. report a novel method involving the combined use of 3D image analysis and transbronchial ICG injection to determine surgical margins for pulmonary sublobar resection in patients with early-stage lung cancer. In their method, preoperative 3D image analysis was accomplished using multi-slice enhanced CT imaging to generate pulmonary angiography and virtual bronchoscopy (18). For each case, these virtual models were then used to run several simulations of anatomical sublobar resection in order to determine the appropriate tumor resection margin.

During surgery, transbronchial injection was used to instill ICG into the target segmental bronchus and distribute it throughout the segmental bronchial tree. Following the generation of virtual bronchoscopy from chest CT imaging, sublobar resection simulation was used to identify the segmental area and margins for resection. Intra-operatively, transbronchial ICG injection yielded well-visualized fluorescence and easily definable segmental borders in 55 of 65 cases (84%). A comparison of preoperative simulated resection and postoperative segmental structure found concordance between preoperative virtual segmentectomy and postoperative segmental structures in 54 of the 58 (93.1%) cases. In a propensity-matched comparison of surgical outcomes between patients who underwent fluorescence-guided sublobar resection with patients who underwent traditional VATS segmentectomy, the analysis showed similar operation length, blood loss, length of hospital stay, and postoperative complications.

## Sentinel Lymph Node Mapping

The use of ICG for intraoperative or postoperative identification of the sentinel node is an exciting area of ongoing research, but its role has not been clearly defined in lung cancer. In cancer, the “sentinel node” refers to the lymph node that receives the initial lymphatic flow from a malignant tumor, theoretically representing the first tissue at risk for malignant dissemination of tumor cells. During a resection for certain types of cancer, if a sentinel node is determined to be negative, a less aggressive lymph node dissection is pursued, limiting morbidity. The regional lymph node status is an important prognostic indicator, particularly in lung cancer. Additionally, patients undergoing a lymph node dissection during pulmonary resection may be upstaged based on nodal status and potentially receive adjuvant therapies. However, in the ACOSOG Z4032 multicenter trial of patients undergoing sublobar resection, 35% of patients were found to undergo resection without lymph node assessment (19). With success in identifying the sentinel node in other malignancies using near infrared imaging, there has been progress in the concept of the sentinel lymph node in lung cancer given that ICG often “lights up” an N1 intrasegmental lymph node.

In 2004, Ito et al. utilized sentinel lymph node navigation using ICG in patients with lung cancer, establishing feasibility and safety (20). Presurgical injection of ICG was administered in peritumoral quadrants in 38 patients undergoing pulmonary lobectomy. During lymph node dissection, a sentinel node was identified by visual inspection in 18% of patients. Additionally, sentinel nodes were derived by relative ICG concentration (1.5 × baseline) in 87.5% of patients with a negative predictive rate of 100%. There was a single false-negative result, which the authors posit was due to tumor obstruction of the primary lymphatic flow.

In 2011, Yamashita et al. evaluated the accuracy of ICG to identify the sentinel lymph node in patients with early stage lung cancer undergoing VATS. Peritumoral injection of ICG was performed and a sentinel lymph node was identified with NIF in 25 of 31 patients (80.7%) with a 0% false-negative rate (21). The noted reasons for failure to identify a sentinel lymph node were attributed to intrapleural adhesions or leakage of ICG contrast after injection.

In 2013, Gilmore et al. reported a dose-escalation clinical trial, evaluating the optimal peritumoral injection of ICG to identify sentinel lymph nodes in human patients with lung cancer (22). In 38 patients, the study revealed that sentinel lymph node identification increased with increased dose-response, finding an 89% success rate at a dose of 1,000 μg of ICG. At this dose, 26 sentinel nodes were identified by near infrared imaging in 15 patients, six of which had metastatic disease on histologic analysis. Metastatic nodal disease was not identified in patients with negative sentinel lymph nodes by ICG evaluation.

Recently, Hachey et al. reported a feasibility study using ICG contrast for tumor and sentinel lymph node localization by ENB (23). Performed in 10 patients with early stage NSCLC, the prospective study revealed that ICG accurately and efficiently identifies the target parenchymal lesion by fluorescent flow, with passive drainage to the sentinel lymph node and regional lymph nodes. The technique identified the sentinel node in eight patients (80%) and the sentinel node status was 100% sensitive for overall nodal staging status. Of note, 12.5% of patients were upstaged based on sentinel lymph node mapping.

In a report reviewing the long-term outcomes after NIF-guided sentinel lymph node mapping with peritumoral ICG injection, Digesu et al. compared patients who underwent mediastinal lymph node sampling with or without sentinel lymph node localization (24). Sentinel lymph node mapping was 100% sensitive and specific for local lymph node status; metastatic disease was detected in the sentinel lymph node in all occurrences (7 patients) of positive lymph node disease (pN+) and not detected in the sentinel node in node negative disease. After a median follow-up of nearly 45 months, the study revealed that patients who underwent sentinel node mapping who were found to have pathologic node negative disease (pN0) had an improved estimated overall and disease-free survival than those who were deemed pN0 by lymph node dissection alone (100 vs. 70%, and 100 vs. 66%, respectively). In the non-sentinel lymph node group, 4 of 15 patients (26%) had recurrence of disease despite pN0 status at resection. The authors attribute part of this outcome to improved overall nodal staging with sentinel lymph node mapping, due to the focused histologic analysis of tumor-specific sentinel nodes, thereby shifting patients previously (incorrectly) deemed pN0 to the correct pN+ status.

## Conclusion

Indocyanine green is emerging as a useful adjunct in the localization of lung nodules and the delineation of the intersegmental plane when given intravenously, via transbronchial injection, or both. There is significant potential for its utility in localizing pulmonary sentinel lymph nodes and its impact on overall and disease-free survival for lung cancer.

## Author Contributions

DF-L, TG, PS, and RC contributed equally to the formation, writing, and editing of this manuscript.

### Conflict of Interest Statement

DF-L discloses compensation for database entry for an unrelated project from Intuitive Surgical. RC discloses relationships with AstraZeneca, Bard Davol, Bovie Medical Corporation, C-SATS, ConMed, Covidien/Medtronic, Ethicon, Fruit Street Health, Google/Verb Surgical, Intuitive Surgical, KCI/Acelity, Myriad Genetics, Neomend, Pinnacle Biologics, ROLO-7, Tego, and TransEnterix. The remaining authors declare that the research was conducted in the absence of any commercial or financial relationships that could be construed as a potential conflict of interest.
